# A Single Cisterna Magna Injection of AAV Leads to Binaural Transduction in Mice

**DOI:** 10.3389/fcell.2021.783504

**Published:** 2022-01-11

**Authors:** Fabian Blanc, Alexis-Pierre Bemelmans, Corentin Affortit, Charlène Joséphine, Jean-Luc Puel, Michel Mondain, Jing Wang

**Affiliations:** ^1^ Institute for Neurosciences of Montpellier (INM), University Montpellier, INSERM, Montpellier, France; ^2^ CHRU Montpellier—Centre Hospitalier Régional Universitaire, Montpellier, France; ^3^ Molecular Imaging Research Center, Institut de Biologie François Jacob, Direction de la Recherche Fondamentale, CEA, Fontenay-aux-Roses, France; ^4^ Université Paris-Saclay, CEA, CNRS, Laboratoire des Maladies Neurodégénératives, mécanismes, thérapies, imagerie, Fontenay-aux-Roses, France

**Keywords:** cisterna magna injection, AAV, cochlea, binaural gene transduction, hearing

## Abstract

Viral-mediated gene augmentation, silencing, or editing offers tremendous promise for the treatment of inherited and acquired deafness. Inner-ear gene therapies often require a safe, clinically useable and effective route of administration to target both ears, while avoiding damage to the delicate structures of the inner ear. Here, we examined the possibility of using a cisterna magna injection as a new cochlear local route for initiating binaural transduction by different serotypes of the adeno-associated virus (AAV2/8, AAV2/9, AAV2/Anc80L65). The results were compared with those following canalostomy injection, one of the existing standard inner ear local delivery routes. Our results demonstrated that a single injection of AAVs enables high-efficiency binaural transduction of almost all inner hair cells with a basal-apical pattern and of large numbers of spiral ganglion neurons of the basal portion of the cochlea, without affecting auditory function and cochlear structures. Taken together, these results reveal the potential for using a cisterna magna injection as a local route for binaural gene therapy applications, but extensive testing will be required before translation beyond mouse models.

## Introduction

Hearing loss is currently the most frequent human sensory deficit and will affect almost one billion people worldwide in 2050 (World Health Organization, 2021). An underlying genetic cause is estimated to be responsible for deafness in 50–60% of affected persons (Angeli et al., 2012). To date, no curative treatment is available for sensorineural hearing loss. The only existing options are sound amplification by hearing aids, and electrical stimulation of auditory nerves *via* cochlear implants. However, the quality of the sound perceived via these two devices still cannot match that of the normal ear (Kronenberger et al., 2014; Illg et al., 2017; Jiam et al., 2017).

During the past decade, promising results have been obtained in cochlear gene therapy of mouse models of genetic deafness (Askew et al., 2015; Isgrig et al., 2017; Pan et al., 2017; Akil et al., 2019; Wu et al., 2021). Recombinant adeno-associated virus (AAV) is the preferred viral vector for gene therapy, due to its non-pathogenicity, low immunogenicity, and long-lasting expression in non-dividing cells (Naso et al., 2017). A major challenge for the delivery of genetic therapeutic materials into the cochlea comes from the inner ear’s anatomical isolation; it is a small, complex structure encased within the densest portion of the temporal bone (Salt and Hirose, 2018). To date, several local routes have been tested to introduce viral vectors into the cochlea (see for review: (Blanc et al., 2020)); one of them is by round-window injection (Chien et al., 2015). After opening the middle ear compartment through the round window membrane, the viral vectors are injected directly in the cochlear perilymph. Recently, Yoshimura and others (Yoshimura et al., 2018) proposed a combined approach that enhances the efficacy of transduction and reduces cochlear damage induced by the puncture of the round window. Indeed, they made an “exit hole” in the posterior semicircular canal (PSCC) before injecting the viral vectors through the round window membrane. This method showed a preferential perfusion flow pattern from the base to the apex in scala tympani without impacting auditory thresholds. Another route is injection into the posterior semicircular canal (PSCC) of the vestibule (Kawamoto et al., 2001), or into the utricle (Lee et al., 2020). These routes allow a high efficiency of transduction of the cochlear and vestibular epithelium (Suzuki et al., 2017; Guo et al., 2018; Tao et al., 2018). Instead of local injections, AAV can also be injected intravenously and reach the inner ear sensory cells efficiently. A recent study (Shibata et al., 2017) showed that intravenous injection of an AAV2/9 carrying the enhanced green fluorescent protein (eGFP) reporter gene resulted in binaural transduction of IHCs, SGNs and vestibular hair cells. However, this systemic route of foreign gene administration requires the intravenous injection of a larger volume of the viral solution, thus increasing the potential of systemic toxicity.

In all the local injections strategies, however, one unilateral injection leads to viral gene transduction only in the injected ear. In addition, opening of the bony labyrinth and the perilymphatic space, and - for round window injection - opening of the middle ear, is mandatory. Disturbance of middle ear sound transduction (Zhu et al., 2016) and of the ionic composition of the perilymph (Mynatt et al., 2006), as well as excessive pressure induced by the injection (Yoshimura et al., 2018) may occur. Taken together, the success of gene therapy still needs a significant and lasting effort to develop safe and clinically useable gene-delivery routes.

The cochlear aqueduct has recently been described as a natural shunt of perilymph away from the cochlea, following perilymphatic injections (Talaei et al., 2019). The cochlear aqueduct is a natural opening in the bony labyrinth, and allows communications between the cochlear perilymph and the cerebrospinal fluid (CSF) (Schuknecht and Seifi, 1963; Salt et al., 2003). It may serve as a pressure-release valve, draining any excess perilymph into the cranial cavity. The Cisterna magna (CM), or posterior cerebellomedullary cistern, collects the CSF produced in the cerebral ventricles. It is located between the cerebellum and the dorsal surface of the medulla oblongata, in proximity to the opening of the cochlear aqueduct. The membrane of the CM can be perforated for CSF collection (Xavier et al., 2018; Manouchehrian et al., 2021). CM injection is a validated technique for CSF-injection of gene transfer vectors in mice (Liu and Duff, 2008; Xavier et al., 2018; Manouchehrian et al., 2021).

In this study to explore the possibility of using CM injection as a new cochlear local delivery route for binaural viral gene transfer, we used AAV2/8, AAV2/9, and AAV2/Anc80L65-expressing eGFP driven by a ubiquitous chicken β-actin (CBA) promoter. Vectors were delivered into adult mice *via* the CM. The efficiency and safety of AAV-mediated gene transfer were evaluated functionally and morphologically, and compared with those from PSCC canalostomy injection. Our results demonstrate for the first time the successful use of CM injection as a novel delivery route for binaural therapeutic gene transfer.

## Methods

### Animals

Male C57BL/6J mice aged 1 month were purchased from Janvier Laboratories (Le Genest Saint Isle) and housed in facilities accredited by the French Ministry of Agriculture and Forestry (C-34-172-36; December 19, 2014). Experiments were carried out in accordance with French Ethical Committee stipulations regarding the care and use of animals for experimental procedures (agreements C75-05-18 and 01476.02, license #6711).

### AAV Production

The AAV-CBA-eGFP construct has been described elsewhere (d’Orange et al., 2018). Recombinant viral particles were generated by transient co-transfection of HEK 293T cells with a three-plasmid system (Berger et al., 2015) with either AAV8, AAV9, or AAVAnc80L65 packaging plasmids. Briefly, 3 days following transfection, viral particles were extracted from cell supernatant and cell lysate, concentrated by PEG precipitation, purified by ultracentrifugation on an iodixaniol step-gradient, and desalted by tangential ultrafiltration. The resulting AAV vector batches were stored at 4°C until further use. AAV concentration was estimated by qPCR using a plasmid of known concentration for the standard curve (Aurnhammer et al., 2012), and is expressed as vector genome per ml (vg/ml).

### Animal Surgery

Mice were anaesthetized with intra-peritoneal injection of zolazepam (40 mg/kg) and xylazine (5 mg/kg). During surgery, their body temperature was maintained at 37.5°C using a heated blanket. After the injection, the animals were closely monitored daily, and no neurological symptoms were observed in the following days. All animals were operated by the same surgeon.

PSCC canalostomy injections were performed as previously described (Guo et al., 2018; Tao et al., 2018). After ensuring that the animal exhibited no reflex to painful stimulation, a left post-auricular incision was made under sterile conditions. Blunt dissection of the sterno-cleïdo-mastoïd muscle exposed the posterior part of the temporal bone and made visible the square angle formed by the lateral and the posterior semicircular canals. Canalostomy of the posterior canal was performed with a 26G bevelled needle and the tip of a polyimitube (microlumen) was inserted toward the crus commune. A small piece of muscle and tissue adhesive (3 M Vetbond, St. Paul, MN) sealed the tube to the temporal bone, avoiding leakage. The polyimide tube was connected to a polyethylene tube, and this to a 30G Hamilton syringe. Injection of 1.5 µL of viral vector (0.5 μL/min) was driven by a micro-pump (Harvard Apparatus), and left in place for 5 min. After removing the tube, the canal was carefully closed by a plug of muscle and tissue adhesive. The skin was closed by 4/0 resorbable sutures. Terramycine (0.04 ml) and Carprofene (0.2 mg/kg) were then administered. The total duration of the procedure was about 20 min. The titer used for AAV2/8, AAV2/9, and AAV2/Anc80L65 was 3.16 × 10^14^ vg/ml, 9.1 × 10^13^ vg/ml and 3.1 × 10^13^ vg/ml, respectively. The chosen titers were based on our pilot study to ensure high level of transduction without compromising hearing functions.

Cisterna magna injection was performed following the same technique as for central nervous system delivery of gene transfer vectors (Liu and Duff, 2008; Xavier et al., 2018; Manouchehrian et al., 2021). Under an operating microscope, an incision was made on the scalp. Median dissection of the muscles of the neck exposed the atlanto-occipital membrane covering the Cisterna Magna. Using a 30G Hamilton syringe, within 30 s, 5 µL of viral vector, or 5 µL of physiological saline, was injected into the Cisterna Magna of AAV-injected or saline-injected mice, respectively. The syringe was left in place for 5 min, and then removed. To avoid leakage, the atlanto-occipital membrane was immediately covered by tissue adhesive. The skin was closed by 4/0 resorbable sutures. Terramycine (0.04 ml) and Carprofene (0.2 mg/kg) were then administered. The total duration of the surgery was about 15 min. For each surgical approach (CM or PSCC canalostomy), for each AAV-serotype and control-saline injected group, three to five mice were used.

### Functional Hearing Assessment

To assess the effect of AAV transduction on hearing function, we recorded the auditory brainstem responses (ABRs) and distortion-product otoacoustic emissions (DPOAEs) before, and 2 weeks after, AAV-injections through both CM and PSCC routes. In addition, five animals of the PSCC group were followed up to 3 months after injection, their hearing function was evaluated once per month by ABR recording (Supplementary Figure S1). All functional evaluations were performed under anesthesia, in a Faraday-shielded, anechoic, sound-proof cage. Body temperature was measured using a thermistor rectal probe, and maintained at 37.5°C ± 1 using a heated blanket.

#### Auditory Brainstem Response

ABR recordings were conducted on anaesthetized mice, and electrodes were placed subcutaneously on the vertex, beneath the ear, and in the back. Tone bursts of 10 ms duration with a 1 ms rise-and-fall time were delivered at a rate of 3/s at 4, 8, 16, and 32 kHz. Sound was delivered by a Tucker-Davis loudspeaker in a calibrated closed-field condition. Amplification of cochlear potentials (20,000) was achieved *via* a Grass P511 differential amplifier, and averaged 1,000 times (Dell Dimensions). Level-amplitude functions of the ABRs were obtained at each frequency (4, 8, 16, and 32 kHz) by varying the level of the tone bursts from 0 to 80 dB sound pressure levels (SPL), in 5 dB incremental steps. ABR threshold was defined as the lowest sound level at which a reproducible waveform could be observed.

#### Distortion-Product Otoacoustic Emissions

DPOAEs were recorded in the external auditory canal using an ER-10C S/N 2528 probe (Etymotic research Inc. Elk Grove Village, IL, United States). The two primary tones of frequency f1 and f2 were generated with a constant f2/f1 ratio of 1.2, and the distortion product 2f1-f2 processed by a Cubdis system HID 40133DP (Mimosa Acoustics Inc., Champaign, IL, United States). The probe was self-calibrated for the two stimulating tones before each recording. f1 and f2 were presented simultaneously, sweeping f2 from 20 to 2 kHz in quarter-octave steps. For each f2 frequency, the distortion product 2f1-f2 and the neighbouring noise amplitude levels were measured and expressed as a function of f2.

### Immunochemistry

Following the last functional hearing assessments 2 weeks after surgery, the mice were sacrificed and their cochleae quickly removed and fixed for 45 min in 4% paraformaldehyde. Immunocytochemistry was performed in cochlear whole-mount preparations. The samples were immunostained with anti-Vglut3 (1/500, Synapic Systems, #135204, RRID: AB_2619825) to label the inner hair cells, and neurofilament (NF 200, 1/500, Sigma Aldrich, AB_477257) to identify the spiral ganglion neurones. Rhodamine-conjugated phalloidin (1:1,000, Thermos Fisher, AB_2572408) was used to label actin. All secondary antibodies were used at a dilution of 1:1,000. These included donkey anti-mouse, anti-rabbit, and anti-guinea pig IgG conjugated to Alexa 488, Alexa 568, or Alexa 664 (Molecular Probes, RRID: AB_141607, RRID: AB_2535792, RRID: AB_2735091). DNA was stained using Hoechst 33,342 (0.002% wt:vol, Sigma). Fluorescent tags were visualized using a confocal microscope (Zeiss 880 Airysc). Direct fluorescence was used to asses for GFP expression, i.e. we did not use any anti-GFP antibodies in our experiments. For IHC cell quantification, control ears were the right - uninjected - ears of the PSCC canalostomy group. No eGFP fluorescence was observed in these cochleae. Images were processed using ImageJ (Wayne Rasband).

### Statistics

Data are expressed as the mean ± SEM. Significant differences between groups were assessed with one-way ANOVA; once the significance of the group differences (*p* ≤ 0.05) was established, Dunn’s tests were used for post-hoc comparisons between pairs of groups. *p* values are indicated in the legends of each figure.

## Results

### A Single CM Injection of Fast Green Led to Green Coloration of Both Cochleae

**FIGURE 1:**
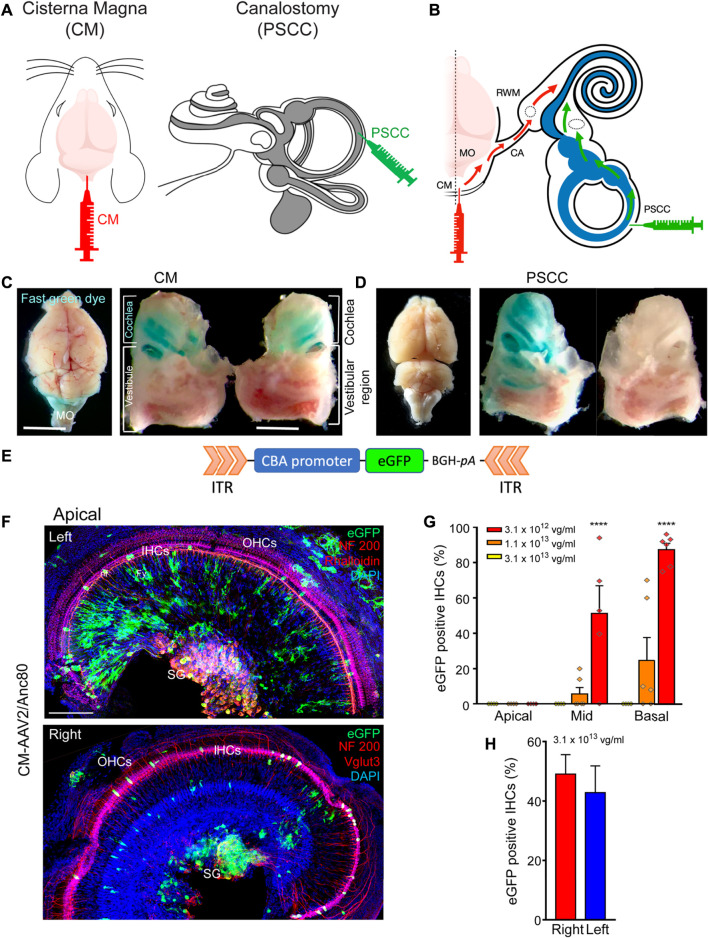
CM injection and cochlear targeting efficiencies. **(A,B)**: Schematics of the surgical approaches for Cisterna Magna (CM) and posterior semicircular canal (PSCC) canalostomy injection **(A)** and diffusion of viral vectors from the CM to the cochlea through the cochlear aqueduct (red arrows) and from the PSCC to the cochlea (green arrows) **(B)**. The grey and white colors in **(A)** indicate the endolymphatic and perilymphatic spaces in the inner ear, respectively. **(C,D)**: Stereomicroscope images of the dissected brain and cochleae following CM injection **(C)** and left PSCC injection **(D)** of green dye. Note the coloration of both cochleae and medulla oblongata (MO in **C**) after CM injection. Note the coloration of the injected cochlea and the vestibule after PSCC injection. **(E)**: Schematic representation of the AAV recombinant genome for AAV2/8, AAV2/9, and AAV2/Anc80L65-CBA-eGFP. CBA: chicken β-actin promoter; eGFP: enhanced Green Fluorescent Protein; BGH-pA: Bovine growth hormone polyadenylation signal. ITR: Inverted terminal repeat. **(F)**: Representative confocal images showing eGFP positive cells in flat-mounted preparations of the apical portion of the left and right cochleae 15 days after CM injection with AAV2/Anc80L65. eGFP positive cells are in green, NF200 immunolabelled auditory nerve fibres (nf, top and bottom images) and spiral ganglion neurons (SG, top and bottom images), Vglut three labelled inner hair cells (bottom image) and Phalloidin-labelled inner and outer hair cells (IHCs and OHCs respectively, top image) are in red. DAPI-labelled nuclei are in blue. Fy: fibrocytes. Scale bar: 50 µm. **(G)**: Transduction efficiency (eGFP positive IHCs) as a function of concentration, at the apical, mid-, and basal regions of the cochlea, 2 weeks after CM injection with AAV2/Anc80L65 (*n* = 3 animals, six cochleae, for each concentration). **(H)**: Average eGFP positive IHCs along the length of the cochlea from the left and right ears following a single CM injection with AAV2/Anc80L65 (*n* = 3 cochleae in each group). Data are expressed as mean ± SEM. One-way ANOVA test was followed by Dunn’s test: ***p ≤ 0.0001, the highest concentration vs*.* the lowest concentration.

To ensure the injected solution *via* the CM route could enter into both left and right cochleae, we firstly compared the coloration of both cochleae following a single CM injection of fast green dye (5 µL of 5% fast green dye in 1X PBS, Figures 1A,B). Our results showed a strong green coloration of the medulla oblongata and the mid- and basal parts of both cochleae (Figure 1C), without green staining of the extreme apical part of the cochleae or the vestibules (Figure 1C). The baso-apical gradient of coloration following CM injection confirmed that the diffusion of the dye in the cochlea was mostly *via* the cochlear aqueduct (Figure 1B). PSCC canalostomy injection (1.5 µL, Figures 1A,B) led to a green coloration only of the cochlea and vestibule of the injected ear, but not of the brain or the contralateral ear (Figure 1D). These results clearly demonstrated the feasibility of two-ear incubation *via* a single CM injection.

### CM Delivery of AAV Allowed Similar Binaural Titer-Dependent IHC Transduction

To assess the cochlear cell-targeting properties, and to define the most efficient viral titer for cochlear gene transfer *via* CM injection, we injected the AAV2/Anc80L65-CBA-eGFP at three different titers: 3.1 × 10^13^ vg/ml, 1.1 × 10^13^ vg/ml, and 3.1 × 10^12^ vg/ml (respectively 1.6 × 10^11^, 5.5 × 10^10^, and 1.6 × 10^10^ genome copies) (Figures 1E–G). We found a viral titer–dependent IHC transduction. The percentage of eGFP-positive IHCs was notably lower with the titer of 1.1 × 10^13^ vg/ml (cochlear base: 24.7 ± 12.9%, middle: 5.7 ± 3.7%, apex: 0.0 ± 0.0%) and was null for the lower titer (3.1 × 10^12^ vg/ml). By contrast, in the basal cochlear region, almost all IHCs were eGFP positive with the higher titer of 3.1 × 10^13^ vg/ml (87.4 ± 3.6%, Figure 1G). Therefore, we chose this concentration for the remaining experiments. The efficiency of the IHC transduction displayed a base-to-apex gradient (3.1 × 10^13^ vg/ml: base: 87.4 ± 3.6, middle: 51.2 ± 15.7%, apex: 0.0 ± 0.0%, Figures 1F,G). More importantly, one CM injection led to a similar transduction of IHCs in both cochleae: 49.0% ± 6.5 and 42.8% ± 9.0 of eGFP positive IHCs along the entire length of the cochlea for the right and left cochleae, respectively (Figures 1F,H).

In addition to IHC transduction, CM injection of AAV2/Anc80L65-CBA-eGFP also efficiently transduced spiral ganglion neurones (SGNs), some auditory nerve-fibre terminals, and fibrocytes in the spiral limbus (Figure 1F).

### Vector- and Route-Dependent Inner Hair-Cell Transduction

**FIGURE 2:**
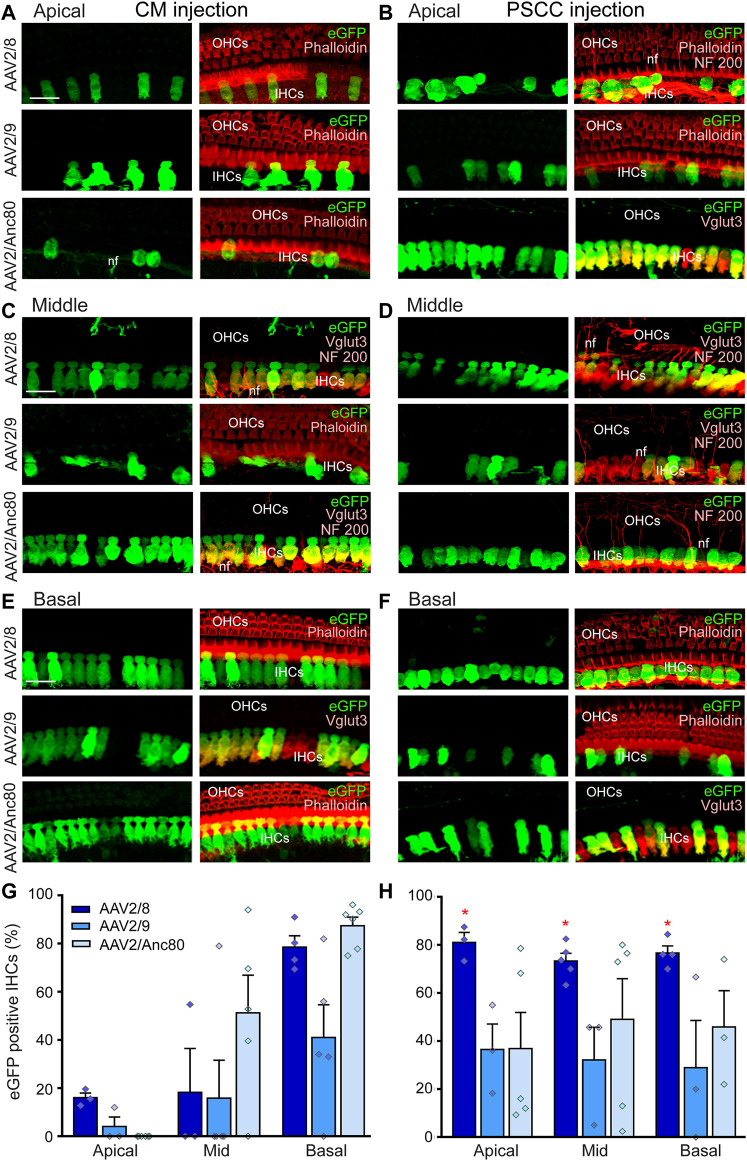
Effects of AAV serotypes on cochlear IHC transduction efficiencies. **(A–F)**: Representative confocal images showing eGFP-positive IHCs in flat-mounted preparations of the organ of Corti of the apical **(A,B)**, middle **(C, D)** and basal **(E,F)** regions of the cochleae 15 days after CM **(A,C,E)** or canalostomy **(B,D,F)** injection with AAV2/8, AAV2/9, or AAV2/Anc80L65. eGFP positive cells are in green, NF200 immunolabelled auditory nerve fibers (nf), Phalloidin-labelled IHCs and OHCs and Vglut3 immunolabelled IHCs are in red. Scale bar: 15 µm. **(G,H)**: Transduction efficiency (eGFP positive IHCs) of the AAV2/8, AAV2/9, and AAV2/Anc80L65 at the apical, mid-, and basal regions of the cochlea, 2 weeks after CM **(G)** or canalostomy **(H)** injection (*n* = 3 to six cochleae for each group). Data are expressed as mean ± SEM. One-way ANOVA test was followed by Dunn’s test: *p ≤ 0.05, AAV2/8 vs*.* AAV2/9 or AAV2/Anc80L65.

Determining the cochlear cell targeted by different AAV serotypes can identify therapeutically relevant AAV capsids for gene delivery to treat hearing disorders *via* CM injection. Here, we assessed the distribution of AAV vector-mediated eGFP expression throughout the cochlea following CM (Figures 2A,C,E) or PSCC injection (Figures 2B,D,F) of three AAV serotypes (AAV2/8, AAV2/9, and AAV2/Anc80L65), all driven by the ubiquitous CBA promoter. We found that the IHCs showed clear evidence of eGFP expression from all of the AAV serotypes, and *via* both routes of injection (Figures 2A–F). We did not find evidence of transduction of the outer hair cells (OHCs) for any serotype or either delivery route (Figures 2A–F).

Quantitative analysis of IHC transduction revealed that the more efficient serotypes following CM injection (Figure 2G) were the AAV2/Anc80L65 (cochlear base: 87.4 ± 3.6%, middle: 51.2 ± 15.7%, apex: 0.0 ± 0.0%) and AAV2/8 (base: 78.5 ± 4.7%, middle: 18.2 ± 18.2%, apex: 16.0 ± 2.0%). AAV2/9 was less efficient (base 41.0 ± 13.6%, middle 15.8 ± 15.8%, apex 4.0 ± 3.1%). This result clearly demonstrated that when injected into the CM, all three AAVs efficiently transduced cochlear IHCs with a baso-apical gradient *in vivo* in adult mice. In the case of the PSCC canalostomy injection, a significantly higher level of the transduction of the IHCs was obtained using AAV2/8 (base: 80.4 ± 7.1%, middle: 73.3 ± 3.2%, apex 81.0 ± 4.1%, *p* < 0.05, AAV2/8 *vs.* using AAV2/Anc80L65 or vs*.* AAV2/9), and there was no baso-apical gradient for any of the three AAV vectors (Figure 2H). The eGFP expression was only observed in the injected ears but not the uninjected contralateral ears in PSCC injection group. These results are consistent with previous reports (Kawamoto et al., 2001; Tao et al., 2018).

Altogether, these results indicate that one single CM injection of AAV leads to binaural IHC transduction, while one canalostomy injection results in single-ear IHC transduction.

### CM Delivery of AAV Allowed the Transduction of the Binaural Spiral-Ganglion Neurones and of the Nerve Fibre Terminals

**FIGURE 3:**
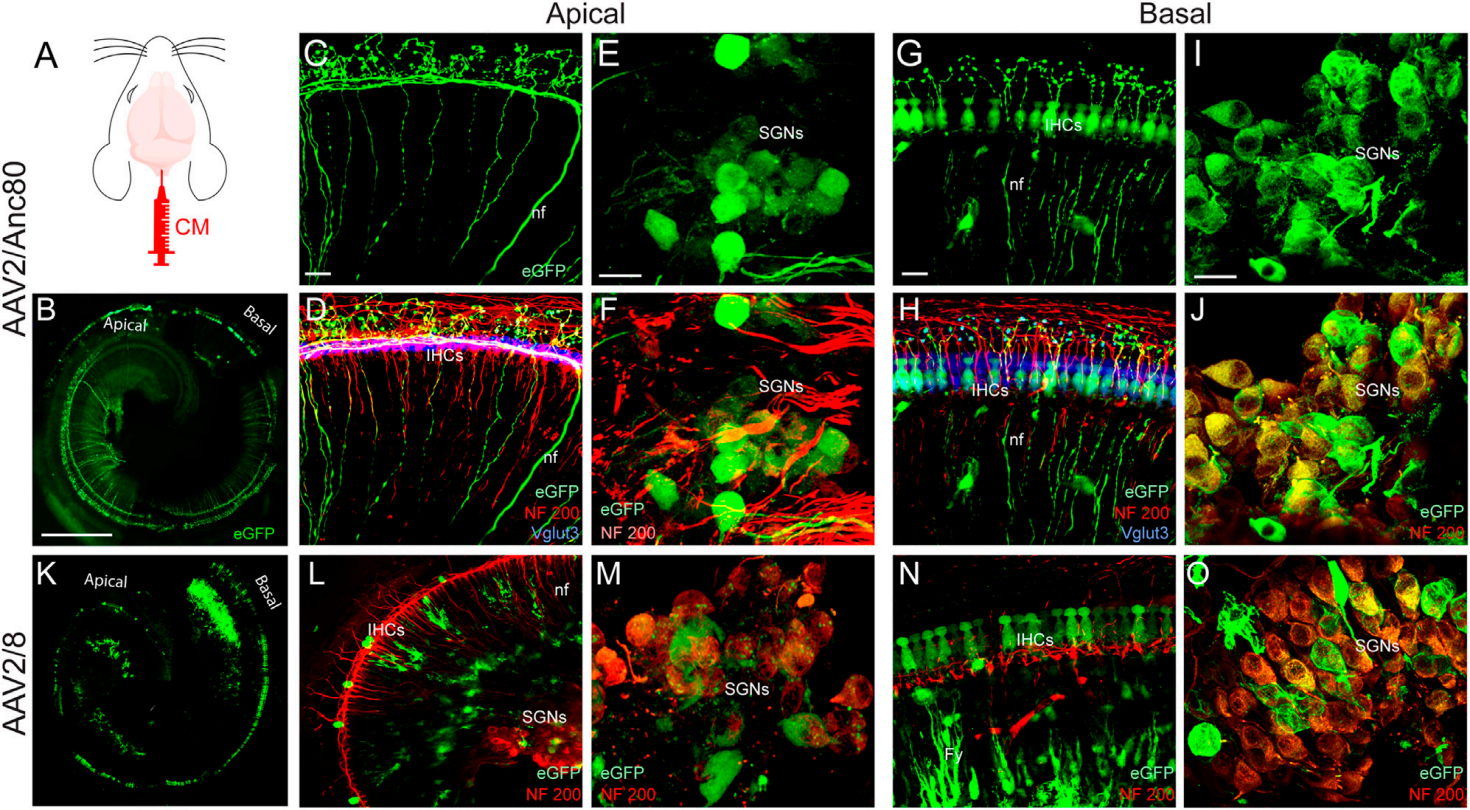
Effects of CM injection of AAV on cochlear neural structure transduction efficiencies. **(A)**: schematization of the CM injection technique. **(B,K)**: Representative mosaic confocal images showing eGFP-positive cochlear structures in whole-mount preparations of cochleae 15 days after CM injection with AAV2/Anc80L65 **(B)** and AAV2/8 **(K)**. Scale bar: 400 µm. **(C,D,G,H,L,N)**: Representative magnification of organs of Corti from the apical **(C,D,L)** and basal regions **(G,H,N)** and the spiral ganglions from the apical **(E,F,M)** and basal regions **(I,J,O)** of the cochleae 15 days after CM injection of AAV2/Anc80L65 **(C–J)** and AAV2/8 (**L–O**). **(C,E,G,I)**: eGFP (green)-positive auditory-nerve fibre terminals (nf, **C,G**), inner hair cells (IHCs, **G**), and spiral ganglion neurons (SGNs, **E, I**). **(D,F,H,J, L–O)**: Merged images. NF200 immunolabelled auditory-nerve fibers and somata of the SGNs are in red. Vglut3 immunolabelled IHCs are in blue. Fy: fibrocytes. Scale bars: 20 µm.

In the cochlea, each IHC is contacted by multiple type-1 SGN axons, each of which follows a radial trajectory terminating at the IHCs and can be specifically labelled by anti-NF200 antibody. OHCs are innervated by type-2 SGNs, whose axons pass the IHC row and take a spiral trajectory, contacting multiple OHCs in the same row. Interestingly, following CM injection, some type-1 and type-2 auditory nerve fibers in the apical and basal cochlear regions were transduced by AAV2/Anc80L65 (Figures 3A–D,G,H). CM delivery of AAV2/Anc80L65 also transduced the SGNs in the apical and basal regions but more importantly in the basal region (Figures 3E,F,I–J). CM injection of AAV2/8 transduced the auditory nerve fibre terminals with less efficiency (Figures 3K–L,N). By contrast, a large number of the SGNs were efficiently transduced with AAV2/8 through CM injection in the apical and basal regions (Figures 3M,O). However, no obvious transduction of the SGNs was observed with AAV2/9 (Data not shown).

### CM Injection did not Affect Hearing or Cochlear Cell Survival

To investigate the potential impact on hearing function of AAV delivery *via* CM injection, we recorded the auditory brainstem responses (ABR), which reflect the synchronous activation of auditory neurones from the cochlea up to the colliculi in response to sound before and 2 weeks after injection. In addition, the distortion-product otoacoustic emissions (DPOAE), reflecting the function of OHCs, were measured at the same times. Our results showed that AAV or control saline injection *via* CM did not affect ABR thresholds or DPOAE amplitude (Figures 3A,B). Similar results were obtained with canalostomy injection (Figures 3C,D). In addition, we did not find any ABR threshold degradation 2 and 3 months after canalostomy injection (Supplementary Figure S1A), which argues in favor of the absence of long-term adverse effects following viral transduction.

**FIGURE 4:**
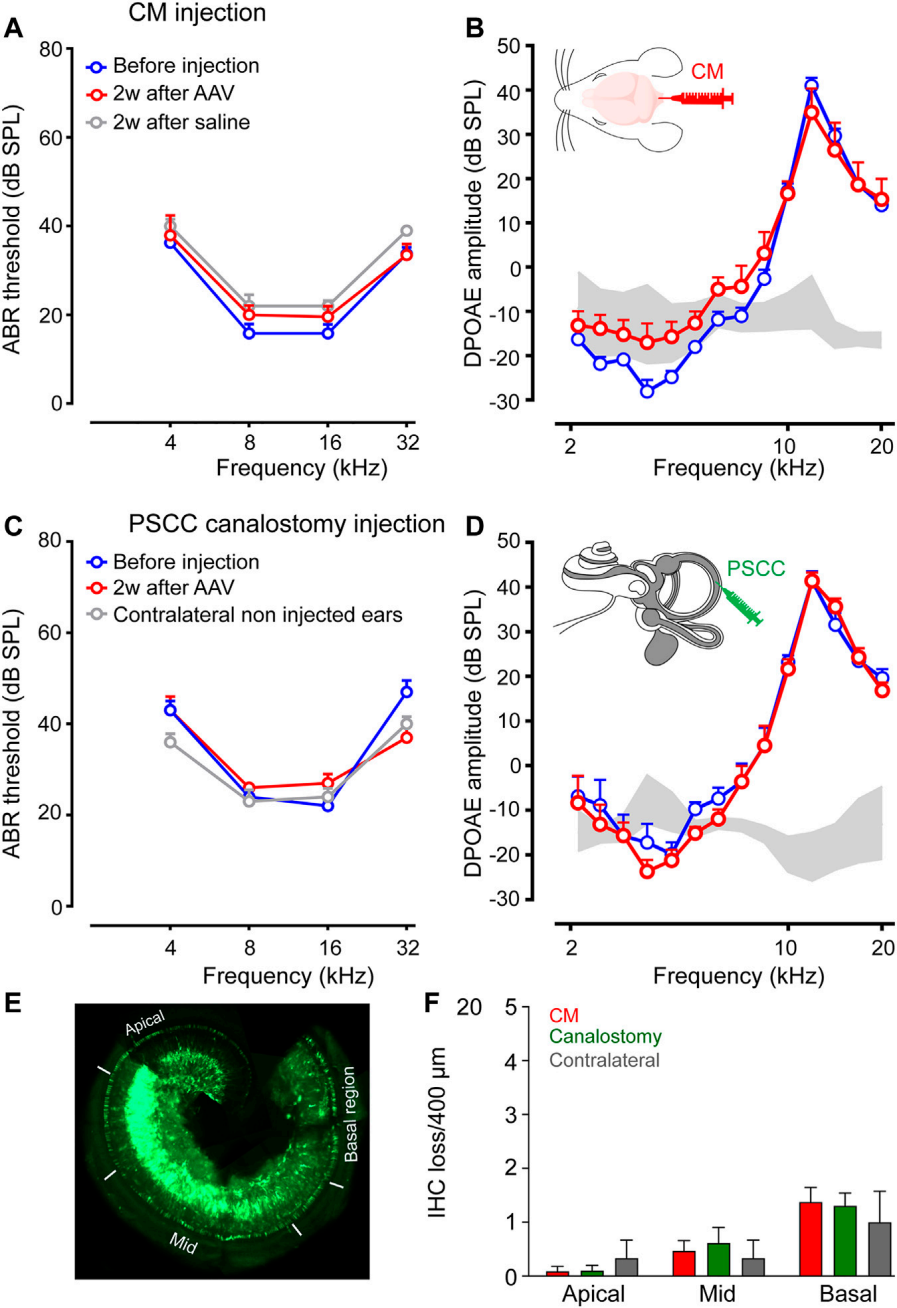
CM and PSCC injection did not alter hearing or cochlear cell morphology. **(A–D)**: Auditory brainstem response (ABR) **(A,C)** and distortion-product otoacoustic emission (DPOAE) amplitudes **(B,D)** recorded before (blue plots) and 2 weeks after AAV injection (red plots), or saline injection (grey plot) *via* Cisterna Magna (CM, **A,B**) or PSCC canalostomy **(C,D)** routes. In the PSCC group, contralateral non-injected ears served as the control (*n* = 5–9 cochleae per group). **(E)**: Representative image showing a whole-mount cochlea 15 days after PSCC injection of AAV2/8. The white lines indicate the apical, mid-, and basal regions in which the counting of the IHC losses were performed. **(F)**: The number of missing cells in the apical, mid-, and basal regions of the cochleae 15 days after CM or PSCC injections or the contralateral non-injected cochleae. Data are expressed as mean ± SEM. One-way ANOVA test.

Consistent with these functional results, surface preparations of uninjected and injected cochleae revealed that losses of OHCs were rarely observed in any conditions. A very slight increase of IHC loss was observed in all injected cochleae. Counting of IHC loss was performed on surface preparations of the organ of Corti (Figure 4E) in which the IHCs were immuno-labelled with anti-Vglut3, and the OHC and IHC from the apical, mid and basal regions were stained with rhodamine-phalloidin conjugate (Supplementary Figure S1B). Our results revealed a negligible and non-significant increase in IHC loss both in CM injected (Basal: 1.4 ± 0.3 IHC loss/400 µm) and in PSCC canalostomy injected (Basal: 0.9 ± 0.3 IHC loss/400 µm) cochleae compared with control, non-injected cochleae (Basal: 0.4 ± 0.7, Figure 4F).

### CM Injection did not Alter Neurological or Vestibular Functions

To avoid the most common reported complication of CM injection in mice (Liu and Duff, 2008), caused by direct damage to the medulla oblongata, we inserted only 1.5 mm of the tip of the needle into the CM. The injected mice were carefully monitored for 2 weeks following surgery. Consistent with a previous report of the central nervous system (Xavier et al., 2018), our results showed that CM injection did not cause any neurological disorders (e.g., disturbance of consciousness, inability to walk, weight loss, wound infection … ) during the 2-week post-operative period.

In addition, we did not observe any specific signs of alterations in vestibular function, such as circling behavior, head tilting, or side-preferences for all injected mice in both CM and PSCC canalostomy groups. In addition, the general behavior of the animals such as their walking and body height was not affected by the surgery, except moderate pain for 2 days. Altogether, both the CM and canalostomy routes seem to be well tolerated by animals. This observation was important in our mind for the future users of CM route for delivering drugs or genes into the inner ear.

## Discussion

### CM Injection and Cochlear Targeting Efficiencies

Due to the anatomical location of the delicate sensory cells surrounded by the dense bony labyrinth, cochlear gene transfer is technically challenging. Intra-cochlear or intra-vestibular injections have been demonstrated as efficient methods of cochlear gene delivery (Askew et al., 2015; Tao et al., 2018). However, there are several shortcomings, including disturbance of the middle ear (Zhu et al., 2016), of cochlear function (Chien et al., 2015; Zhu et al., 2016), and excessive pressure (Salt and DeMott, 1998; Yoshimura et al., 2018), or lack of specificity of the targeted cells (Lee et al., 2020; Wu et al., 2021). In this study, we investigated the diffusion of the three most efficient and commonly used AAV vectors for cochlear gene transfer (AAV2/8, AAV2/9, and AAV2/Anc80L65) following CM or PSCC delivery. The CSF in the CM communicates with the perilymph of the inner ear *via* the cochlear aqueduct in mammals (Salt and Hirose, 2018). Physiologically, a small volume of CSF (30 nL/min) enters the basal portion of the cochlea in guinea pigs (Salt et al., 2015). A recent study of dye tracking following round-window membrane injection and PSCC canalostomy injection in mice showed that the cochlear aqueduct dictates dye diffusion in the inner ear, by ensuring a pressure shunt to the cranial cavity (Talaei et al., 2019). In our study, we demonstrated the proof of concept that this shunt can be used in reverse (i.e. from the cranial cavity to the cochlea) to transduce the cochlear sensory IHC and SGN.

Consistent with previous studies (Kawamoto et al., 2001; Suzuki et al., 2017; Guo et al., 2018; Tao et al., 2018), we showed that PSCC canalostomy injection resulted in efficient transduction of IHCs without a baso-apical gradient, and to a lesser extent, of some SGNs in the injected ear. Interestingly, a single CM injection of all three AAVs resulted in a bilateral transduction of IHCs, with a base-to-apex gradient. This gradient of transduction is consistent with a diffusion of viral vectors from the cochlear aqueduct to the apex *via* the scala tympani. The baso-apical gradient was also found by Chien et al. (2015) after round-window membrane injection of AAV2/8, and probably resulted from the direction of the injection of AAV into the base of the cochlea. In our study, CM injection led to a significant gradient of transduction from basal to apical IHC. This pattern could be highly relevant for treating high-frequency hearing disorders involving dysfunction of basal hair cells, such as DFNA2 (Kharkovets et al., 2006; Carignano et al., 2019), DFNA3 (Smith and Ranum, 1993), DFNA5 (Laer et al., 1998), DFNA9 (Robertson et al., 1998), and DFNA17 (Lalwani et al., 2000).

In addition to IHC transduction, CM injection targeted a large number of spiral ganglion neurons and auditory nerve fibers along the cochlea, with both AAV2/Anc80L65 and AAV2/8. The efficient transduction of SGN by CM injection could, in part, be driven by diffusion along the cochlear nerve in the modiolus, independent of diffusion along the scala tympani. Anatomically, from its emergence from the brainstem to the most distal part of the modiolus, the cochlear nerve is surrounded by CSF (Salt and Hirose, 2018), and is thus in direct contact with viral vectors injected into the CM. Future dye-tracking studies would be interesting, to investigate how, and in which proportion, the modiolus facilitates the passage of viral vectors to the SGN in CM injection.

Consistent with other studies (Shu et al., 2016; Landegger et al., 2017; Tao et al., 2018), we did not observe transduction of OHC in adult mice with the three AAV capsids that we tested, *via* either CM and PSCC canalostomy injection. Suzuki et al. (Suzuki et al., 2017) showed that AAV2/Anc80L65 induced a strong transduction of OHC in adult cochleae after PSCC canalostomy injection, whereas we did not find transduction of OHCs with this serotype. One of the explanations of this contradicting finding could be the different promotors used in their study and ours. Although both vectors were derived from the AAV2 genome and contain a Woodchuck Hepatitis Virus Regulatory Element (WPRE) to boost transgene expression, Suzuki et al. (Suzuki et al., 2017) used a CMV promoter, whereas our construct contained a CBA promoter.

### Effects of AAV Serotypes on Cochlear Cell-Transduction Efficiencies

In this study, to probe the possibility of using CM injection as a new cochlear local route for binaural transduction we tested AAV2/8, AAV2/9, and AAV2/Anc80L65. The transduction profile of the first two is already well defined in the cochlea (Isgrig et al., 2017; Landegger et al., 2017; Tao et al., 2018). The last one, a synthetic serotype (Zinn et al., 2015), displays better transduction properties in several neuro-sensory tissues and the central nervous system than any previously identified AAV (Wang et al., 2017; Carvalho et al., 2018; Hudry et al., 2018). In line with these previous studies, our results attest that AAV2/Anc80L65 is the most efficient vector for IHC transduction *via* CM injection. In addition, among the three tested capsids, Anc80L65 induced strong transduction in the SGNs and the nerve fibre terminals following CM injection.

### Binaural Cochlear Gene Delivery Without Imperilling Hearing, Vestibular, or Neurological Functions

Distanced gene delivery into the cochlea through the utricle or PSCC canalostomy injection (Kawamoto et al., 2001; Lee et al., 2020) seems to be a promising option for cochlear gene transfer to avoid injection-related trauma by removing the injection site from the cochlea. In addition, these more distant delivery routes may still drive a high-efficiency transduction of the cochlear and vestibular sensory hair cells (Suzuki et al., 2017; György et al., 2019), their supporting cells (Wang et al., 2014; Isgrig et al., 2019), and SGN (Suzuki et al., 2017).

Here, following CM and PSCC injection, we did not observe any neurological disorders (e.g., disturbance of consciousness, inability to walk, weight loss, wound infection … ) or vestibular disorders (circling behavior, head tilting, or side-preferences). In addition, the hearing function assessments showed that AAV injection *via* both routes did not affected ABR thresholds or DPOAE amplitudes. These functional results were then confirmed by hair-cell counts. Our results reveal the potential for using CM injection as a safe local route for binaural AAV-mediated gene therapy applications in mice. Compared with all existing local routes, the advantages of the CM injection route are: 1) no opening of inner-ear liquid compartments is needed, thus preventing inner-ear anatomical and functional disorders; 2) it results in binaural gene transduction with a single injection. Our results demonstrated that AAV vectors injected into the CSF cross the cochlear aqueduct to reach the cochleae of both ears. This delivery route may also be of interest for delivering other therapeutic materials such as non-viral particles, drugs, and/or ototoxic molecules into the two ears.

### Potential Future Human Application and Challenges

In humans, the CM is an established route used for CSF collection and drug injection into the central nervous system (CNS) (Ayer, 1920; Lutters and Koehler, 2020). Recently, it has been proposed as an efficient route for CNS gene therapy in non-human primates (Hinderer et al., 2014). More importantly, a recent pioneering study in two Tay-Sachs patients showed the safety and feasibility of AAV9 gene transfer to the CNS through CM infusion (Taghian et al., 2020). In this study, the AAV vector was successfully delivered to the CM through adaptation of an intravascular microcatheter without any complications.

For translational cochlear gene transfer following viral vector infusion into the CM, the issue of opening of the cochlear aqueduct must be raised. The role of the cochlear aqueduct is unclear, but it may serve as a pressure regulator of the inner ear (Carlborg et al., 1982). Indeed, while the cochlear aqueduct is constantly open in small mammals (e.g., guinea pig (Salt et al., 2012) and mouse (Talaei et al., 2019)), it seems only to be open in 34% of patients. Instead, it is partially filled with connective tissue in 59%, and totally obstructed in 7%, as attested by a post-mortem study of 101 temporal bones (Gopen et al., 1997). High-resolution Magnetic Resonance Imaging (MRI) is a precise tool to explore diffusion of Gadolinium in the inner ear fluid compartments. Nakashima et al. (Nakashima et al., 2012) showed that Gadolinium injected into the inner ear moves into the CSF *via* the internal auditory meatus. However, pathological situations inform us of the possibility of passage for viral and bacterial pathogens, and even erythrocytes, between the inner ear and CSF (Holden and Schuknecht, 1968; Marsot-Dupuch et al., 2001). Such passage is much more common in infants, possibly because of the shorter length of the cochlear aqueduct (Palva, 1970). More precise data based on MRI, taking into account the size of AAV particles, could be relevant in the future. Moreover, strategies to improve viral passage through a cochlear aqueduct filled with connective tissue, such as by transiently loosening the extracellular matrix (Dalkara et al., 2009), could be explored, with careful monitoring of potential neurotoxicity.

Local injections, and much more distant-site injections, expose patients to the risk of off-target side effects. Communications between the cochlear perilymph and the systemic circulation include: the cochlear aqueduct, the cochlear vascularization, the bone marrow surrounding the bony labyrinth, the round and oval windows, and the modiolus (for review see Salt and Hirose (Salt and Hirose, 2018)). Since we chose to focus on inner-ear genetic transduction, we did not look for eGFP expression in the brain. However, a similar study using CM injection of AAV9 described strong transduction of meninges, cerebellum, cerebral cortex, and medulla oblongata in non-human primates (Hinderer et al., 2014). Infusion into the CSF conventionally led to transduction in the brain in mice and rats (Chatterjee et al., 2021; DeRosa et al., 2021; Francis et al., 2021). To transfer these surgical approaches of the inner ear into clinical practice, progress will have to be made to set up molecular strategies to avoid off-target side effects. Ubiquitous promoters, such as CBA, PGK, or CMV open the risk of the unwanted transduction of structures surrounding the cochlea, in particular those in the central nervous system. Defining a cochlear-specific AAV serotype or promoter could limit the effect of a transgene specifically to the targeted cochlear cells.

## Data Availability

The raw data supporting the conclusion of this article will be made available by the authors, without undue reservation.
