# Supplementary material for: A Single Cisterna Magna Injection of AAV Leads to Binaural Transduction in Mice
Source: Front Cell Dev Biol. 2022 Jan 11;9:783504. doi: 10.3389/fcell.2021.783504 (PMC8787364; doi:10.3389/fcell.2021.783504)
Supplement: Supplementary file 1 [file DataSheet1.docx]

**Additional file 1**

**Supplemental Data for**

**A single cisterna magna injection of AAV leads to binaural** **transduction in mice**

Fabian Blanc^1,2^, Alexis-Pierre Bemelmans^3,4^, Corentin Affortit^1^, Charlène Joséphine^3,4^, Jean-Luc Puel^1, *^, Michel Mondain^1,2, *^ , Jing Wang^1,2^

**Figure S1**

**
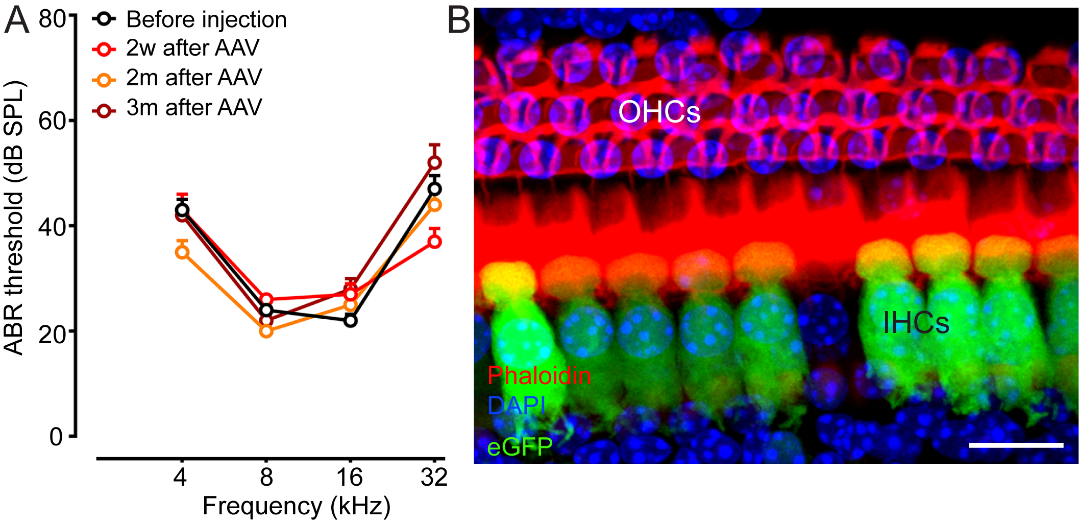
**

**Figure S1. Hearing assessments and hair cell counts after AAV injection**

**A:** ABR thresholds recorded before surgery (black plots) and 2 weeks (red plots), 2 (yellow plots) and 3 (dark red plots) months after posterior semicircular canal (PSCC) canalostomy injection (n=5 mice, AAV2/8-CBA-eGFP). **B:** a flat preparation of basal portion of the cochlea follow PSCC injection with AAV2/8 and labelled with Phalloidin for IHC counting. eGFP positive cells are in green, Phalloidin-labelled IHCs and OHCs are in red and DAPI labelled nuclei are in blue. Scale bar: 10 µm.
